# Enhancement of cytotoxic and antioxidant activities of *Digenea simplex* chloroform extract using the nanosuspension technique

**DOI:** 10.1007/s00449-022-02833-6

**Published:** 2022-12-20

**Authors:** Hanaa M. El-Rafie, Enas A. Hasan, Magdy K. Zahran

**Affiliations:** 1 Pharmacognosy Department, Pharmaceutical and Drug Industries Research Institute, National Research Centre, 33 El Bohouth St. Former El-Tahrir St., Dokki, P.O. 12622, Giza, Egypt; 2 The Holding Company of the Biological Products and Vaccines, 51 Wezaret Al Zeraa St., Agouza, Giza, Egypt; 3 Chemistry Department, Faculty of Science, Helwan University, Ain-Helwan, Cairo, 11795 Egypt

**Keywords:** Nanosuspension, Chloroform extract, *Digenea simplex*, Antioxidant, Cytotoxicity

## Abstract

*Digenea simplex* (*D. simplex*), an Egyptian marine red macroalga, contains a diverse group of phytochemicals with unique bioactivities. At the same time, the synthesis of nanosuspension (NS) has received increasing interest to optimize the technological aspects of drugs. Thence, the main objective of this work was to use the chloroform extract (ChlE) of *D. simplex* to prepare its nanosuspension (ChlE-NS) formulation to increase its aqueous solubility, thereby improving its bioactivity. By using FTIR, GC/MS analysis, and phytochemical screening assays, the chemical profiling of ChlE was assessed. NS was prepared by the antisolvent precipitation technique using 1.5% w/v polyvinyl alcohol (PVA). A light microscope, FTIR, particle size distribution, polydispersity index (PDI), and zeta potential (ZP) measurements was used to characterize the prepared NS. Four cancer cell lines were used in the MTT experiment to investigate the anticancer potential of ChlE and ChlE-NS. An apoptotic mechanism was established using acridine orange/ethidium bromide (AO/EB) dual staining, DNA fragmentation, and increased caspase activity. ChlE and ChlE-NS were also evaluated as antioxidants using DPPH and ABTS free radical assays. The results showed that, when compared to ChlE, ChlE-NS had greater cytotoxic activity against the four cancer cell lines. However, results of antioxidant activity showed that ChlE-NS had an IC_50_ of 36.86 ± 0.09 and 63.5 ± 0.47%, while ChlE had values of 39.90 ± 0.08 and 86.5 ± 0.8% in DPPH and ABTS assays, respectively. Based on the results of this research, *D. simplex* ChlE-NS may be an effective strategy for enhancing ChlE's cytotoxic and antioxidant activities.

**Graphical abstract:**

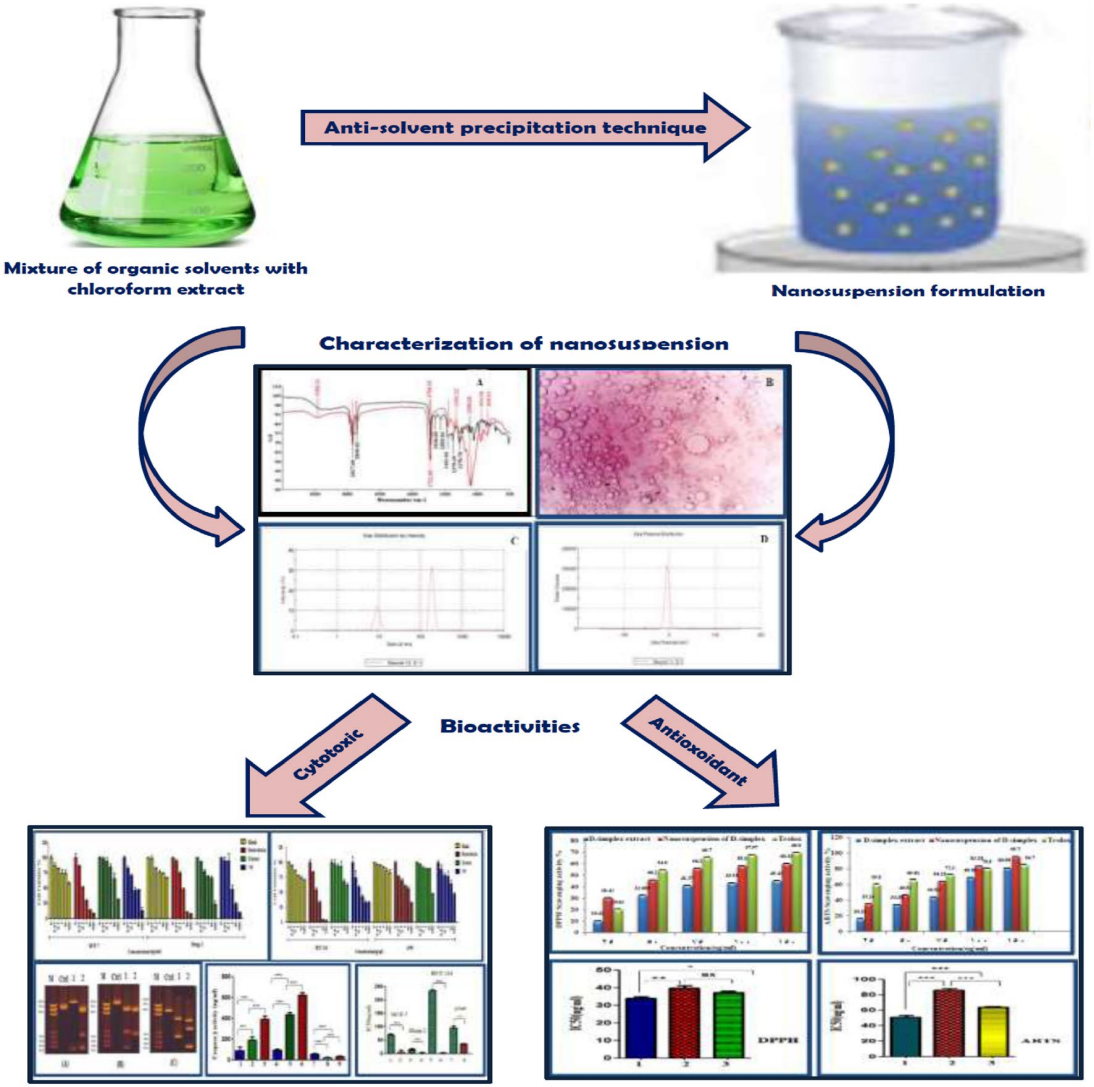

## Introduction

As a part of nanotechnology, nanosuspension technology has emerged as a fantastic field in the pharmaceutical industry [1–3]. Nanosuspension techniques are often used to make drugs and extracts that do not dissolve well in water dissolve better [4]. This allows therapeutic goals to be met and the active drug to be delivered at the best rate and amount [5–7]. Nanosuspensions are colloidal dispersions of extracts and drugs nanoparticles stabilized by surfactants, which are poised to provide a slew of new approaches and methods for modernizing pharmacological and therapeutic preparations [8]. Phytomedicines, in general, due to their lower side effects and toxicity than drugs of synthetic origin, are used all over the world [9–11]. Of these phytomedicines, marine macro-algal nanosuspension formulations open a new era in drug discovery [12]. Some benefits of nanosuspensions include bioavailability, improved stability, passive targeting of drugs, and solubility. This is important because one of the biggest problems in the pharmaceutical industry is the difficulty of dissolving in water, which limits how well drugs can be delivered. Nanosuspensions can also change the drug's pharmacokinetics, which improves the drug's effectiveness and safety.

The vastness of the seas and oceans accounts for 99% of the total living space on Earth [13]. Seas and oceans are also comprised of extraordinary environmental diversity, from salinities, pressures, and temperatures; from bright light to no light; anoxic to normoxic; heavy metals and chemicals; anthropogenic pollutants; and allelopathic defenses. Based on this environmental diversity, the earth’s marine-dwelling inhabitants constitute a plethora of diversified resources for novel drugs to battle various types of human diseases [14–16].

From ancient times until now, marine macro-algae have been used to treat many diseases, either as pure compounds or as standardized extracts. This has led to the development of new drugs. Marine macroalgae are rich in chemical constituents with potential pharmacological effects such as flavonoids, phenolics, polysaccharides, alkaloids, pigments, organic acids, tannins, and terpenes [17–20]. These phytochemicals contain antioxidant substances recognized for their capacity to protect somatic cells from the damaging effects of reactive oxygen species (ROS) [21, 22]. ROS can be produced either endogenously within the human body as a result of metabolic activity or exogenously as a result of radiation, cigarette smoking, ozone, certain foods, drugs, or environmental pollutants [22, 23]. ROS are stabilized by processes that damage cells and form cancer-causing DNA adducts [24]. ROS are the leading cause of cardiovascular disease, ageing, and cancer in humans, and it may be minimized by increasing the use of antioxidants [25]. In addition, recent studies have shown that marine-macroalgal phytoconstituents could be powerful anticancer agents [26–28]. These anticancer agents are the most effective treatments for cancer and are not toxic to normal cells. The cytotoxic and antioxidant effects of *D. simplex* chloroform extract have received insufficient attention, and published data on this species are mostly from waters other than the Egyptian Red Sea beaches [29, 30]. It is also the first time a nanosuspension formulation based on this extract has been developed. So, the goal of this study was to investigate the antioxidant and anticancer properties of the chloroform extract of the macroalga *D. simplex* from the shores of Egypt, as well as how the development of the nanosuspension formulation could improve these bioactivities.

## Materials and methods

### Chemicals

Polyvinyl alcohol (PVA) and 2,2-diphenyl-1-picryl-hydrazyl-hydrate (DPPH)^**·**^ of analytical grade were purchased from Sigma-Aldrich (St. Louis, MO). Tween 40 was purchased from Fisher Chemical Company (USA). The HCT116, HEPG2, MCF7, and A549 cell lines were purchased from the American Type Culture Collection (ATTC, Manassas, VA, USA). 2,2-Azino-bis (3-ethylbenzthiazoline)-6-sulfonic (ABTS) and Trolox were purchased from Sigma Chemical Co. (Madrid, Spain). Other chemicals were of analytic purity and were used without further purification.

### Collection and identification of algal samples

The alga was collected by scuba diving on the Red Sea beach, Sharm EL Shaikh, Sinai, Egypt, between September 2019 and May 2020. To remove any foreign materials, the freshly collected alga was thoroughly rinsed with both sea and tap water. Prof. Dr. Rawheya Salah El-Din, Professor of Botany, Faculty of Science, and Dr. Ehab El Belely, Ph.D., Applied Phycology, Botany and Microbiology Department, Faculty of Science, Al-Azhar University, identified it as *Digenea simplex* (Wulfen) C. Agardh. Five hundred grams of this alga were dried in shade and powdered with a grinder.

### Preparation and characterization of ChlE

After grinding, the alga was extracted with petroleum ether (60°–80°) and left to defat for 10 h. It was then extracted in a Soxhlet with chloroform until the extraction was complete. ChlE was filtered, dried at reduced pressure using a Buchi vacuum rotary evaporator (Model R-200), weighed, and put away for future investigation.

#### Phytochemical screening

Several phytoconstituents in *D. simplex* ChlE were qualitatively screened using previously published chemical analyses [31, 32]. The results are tabulated in Table 1.

**Table 1:** Phytochemical screening of *D. simplex* ChlE

Phytochemical constituents	Results
Carbohydrates (reducing sugars)	+
Saponins	−
Terpenoids	+ + +
Sterols	+
Flavonoids	–
Phenols	+ + +
Tannins	–
Cardiac glycosides	–
Proteins/amino acids	+
Alkaloids	+
Anthraquinones	–

+ Present, – Absent

#### Gas chromatography–mass spectrometry (GC/MS) analysis of ChlE

Quantitative analysis of the phytoconstituents of *D. simplex* ChlE was performed using a capillary column made of fused silica (5% phenyl methyl polysiloxane), 30 m length, 0.25 mm I.D. and 0.25 µm thickness, DB-5, with helium as the carrier gas at 13 psi, oven temperature 50–280 °C, chart speed 0.5 cm/min, ion source temperature 220 °C, ionization voltage 70 eV, accelerated voltage 2000 v, and volume injected 1 µL. For identification purposes, the retention times and mass spectra of the obtained data were compared with those of the library (Wiley International, USA), NIST (National Institute of Standards and Technology, USA), and/or published data to ensure that they were obtained from the correct sources [33]. Table 2 lists the name, base peak, retention time, and molecular weight of each extracted ingredient.

**Table 2:** Compounds identified in *D. simplex* ChlE by GC–MS

No	Name of compound	*R*_t_	BP	*M*^+^	MF	Area%
I. Straight chain hydrocarbon						
1	Heptadecane	26:47	57	240	C_17_H_36_	0.58
2	Nonadecane	30:66	57	268	C_19_H_40_	0.39
3	Octacosane	37:68	57	394	C_28_H_58_	0.97
4	Nonacosane	39:46	57	408	C_29_H_60_	1.25
5	Triacontane	41:04	57	422	C_30_H_62_	1.84
6	Hentriacontane	43:15	57	436	C_31_H_64_	3.00
7	Dotriacontane	45:38	57	450	C_32_H_66_	4.19
8	Tritriacontane	48:03	57	464	C_33_H_68_	3.78
9	Tetratriacontene	50:05	55	476	C_34_H_68_	1.90
10	Tetratriacontane	51:76	57	478	C_34_H_70_	3.90
11	Hexatriacontane	53:18	57	506	C_36_H_74_	0.81
Total identified straight chain hydrocarbon 22.61						
II. Branched hydrocarbons						
12	Methyl octadecane	29:44	57	268	C_19_H_40_	0.36
13	5-Methyl-5-docosene	32:19	55	322	C_23_H_46_	0.31
14	Methyldocosane	33:27	57	324	C_23_H_48_	0.66
15	13-Methylheptacosane	36:15	57	394	C_28_H_58_	0.51
16	Methylhentriacontane	43:99	57	450	C_32_H_66_	4.01
17	Methyldotriacontane	46:73	57	464	C_33_H_68_	4.30
18	Methyl-tritriacontane	49:30	57	478	C_34_H_70_	3.17
Total identified branched hydrocarbons 13.32						
III. Oxygenated hydrocarbons						
A. Esters						
19	Diisobutyl phthalate	29:74	149	278	C_16_H_22_O_4_	1.16
20	Methylhexadecanoate	31:10	74	270	C_17_H_34_O_2_	0.74
21	Butyl hex-3-yl phthalate	31:61	149	306	C_18_H_26_O_4_	1.85
22	Ethylnonadecanoate	32:39	88	326	C_21_H_42_O_2_	0.58
23	2,3-Dihydroxypropyl (*Z*)-octadec-9-enoate	35:13	55	356	C_21_H_40_O_4_	8.07
24	Tributyl acetylcitrate	36:29	185	402	C_20_H_34_O_8_	0.73
25	Didecan-2-yl phthalate	41:45	149	446	C_28_H_46_O_4_	2.69
Total identified esters 15.82						
B. Ketones						
26	Nonalactone	9:16	99	156	C_9_H_16_O_2_	0.41
27	4-Hydroxydecan-5-one	17:83	69	172	C_10_H_20_O_2_	4.55
Total identified ketones 4.96						
C. Acids						
28	Tetradecanoic acid	27:76	73	228	C_14_H_28_O_2_	1.03
29	Octadecadienoic acid	31:40	81	280	C_18_H_32_O_2_	1.40
30	9-Octadecenoic acid	31:88	55	282	C_18_H_34_O_2_	16.47
31	Tricosanoic acid	33:85	73	354	C_23_H_46_O_2_	0.66
32	Lignoceric acid	35:48	73	368	C_24_H_48_O_2_	1.59
Total identified acids 21.15						
D. Sterols						
33	Cholesterol benzoate	49:86	105	490	C_34_H_50_O_2_	3.57
34	Fucosterol	52:35	314	412	C_29_H_48_O	2.67
Total identified sterols 6.24						
IV Terpenoidal compounds						
35	Squalene	45:53	69	410	C_30_H_50_	0.68
36	Neophytadiene	29:33	95	278	C_20_H_38_	0.41
Total identified terpenoidal compounds 1.09						
V. Miscellaneous compounds						
37	Crotetamide	28:15	69	226	C_12_H_22_N_2_O_2_	1.13
38	6-Tetradecanesulfonic acid, butyl ester	34:39	67	334	C_18_H_38_O_3_S	0.38
39	2-Chloroethyl linoleate	34:93	67	342	C_20_H_35_ClO_2_	0.78
	Total miscellaneous compounds					2.29
Total Identified Constituents 87.48						

### Formulation and characterization of ChlE-NS

With a simple modification of a previously published process [34], the antisolvent precipitation technique was used to prepare NS. Using high-frequency sonication for 70 s, 1.5 g of ChlE was dissolved in 12 mL acetone and ethanol (3:1). With continuous magnetic stirring at 1000 rpm, the resulting solution (1 mL min^−1^) was progressively injected into 20 mL aqueous solution containing 1.5% (w/v) PVA using a syringe. The resulting emulsion was diluted with 50 mL PVA solution (0.2% w/v in water) to produce the desired consistency. Following that, 5 mL tween 40 was added while the mixture was continuously stirred at 500 rpm at room temperature for 6 h to allow for solvent evaporation and the production of nanoparticles. Fourier transform infrared spectroscopy (FTIR) study of ChlE and ChlE-NS was done using a Japanese-made FTIR spectrometer, the 6100 JASCO, in the IR region of 500–4000 nm. In addition, Malvern Instruments' Zetasizer Nano ZS, Malvern, UK, was used to assess particle size, particle size distribution, and polydispersity index (PDI) using Dynamic Light Scattering (DLS). At 25 °C and a count rate of 254.1 kcps, double-distilled water was utilized as a dispersant, with a dielectric constant, viscosity, and refractive index of 78.5, 0.8872 cP, and 1.330, respectively. After diluting it, the sample was analyzed at the appropriate concentration.

### Biological activities

#### Cytotoxic activity

##### Cell lines

The human colon cancer cell line HCT 116 (ATCC^®^ CCL-247^™^), human liver cancer HEPG2 cell line (ATCC^®^ HB-8065^™^) cell lines, human breast cancer MCF7 cell line (ATCC^®^ HTB-22^™^), human lung cancer A549 cell line (ATCC^®^ CCL-185^™^), and normal human lung fibroblast WI-38 (ATCC^®^ CCL-75^™^) were purchased from the American Type Culture Collection (Manassas, VA, USA). Normal human lung fibroblast cells and cancer cell lines were cultured in Dulbecco medium supplemented with 10% heat-inactivated (56 °C) foetal bovine serum, recommended RPMI-1640, penicillin (100 IU/mL), l-glutamine (3 mM), streptomycin (100 mg/mL), and 25 mM 4-(2-hydroxyethyl)-1-piperazine ethanesulfonic acid (HEPES). An incubator atmosphere of 37 °C, 95% air, and 5% CO_2_ was used for the cells to grow [35].

##### Treatment of cell lines

In dimethyl sulfoxide (DMSO), stock solutions (10 mg/mL) of the extract and nanoform were dissolved, and the needed concentrations were produced by dissolving the stock solutions in the appropriate medium. For 24 h, five thousand cancer and normal cells were plated on a flat-bottomed microplate (96-well) and allowed to colonize. In the experiment, cells were exposed to several doses of ChlE and ChlE-NS: 100, 10, 1, 0.1, 0.01 g/mL, and 0 for control wells, where cells were just treated with the nutritive medium for 48 h. Cell survival was determined using the MTT viability test, as indicated by van Meerloo et al. [36] with some changes, 72 h after the addition of ChlE and ChlE-NS. In short, each well received 50 mL MTT solution [5 mg/mL in phosphate-buffered saline (PBS)]. The samples were then incubated for a further 4 h. After that, 100 μL DMSO was added, resulting in the formation of a purple formazan (insoluble product) as a consequence of MTT dye conversion by viable cells, as well as a yellow colour that came from dead cells. The number of viable cells in each well was proportional to the intensity of light absorbance, which was measured at 570 nm using an ELISA (an enzyme-linked immunosorbent assay) test plate reader by Biotek (ELX-800) [37]. To calculate the percentage of cell survival, the absorbance of a sample (*A*_sample_) of cells grown in the presence of different concentrations of the examined extracts and nanoform was divided by the control optical density (*A*_control_) of cells grown solely in nutritional medium and multiplied by 100 (Eq. 1). The half-maximal inhibitory concentration (IC_50_%) (Eq. 2) was defined as the concentration that inhibited 50% of the enzyme as compared to a vehicle-treated control. All experiments were conducted in triplicate and the results are presented as mean standard deviation (± SD).

1 $$\mathrm{Cell \,survival }\left(\mathrm{\%}\right)=\left[\frac{{A}_{\mathrm{sample}} - {A}_{\mathrm{control}}}{{A}_{\mathrm{control}}}\right]\times 100,$$

2 $${\text{IC}}_{{{50}}} \left( \% \right) = \left[ {\frac{{{\text{OD of maximal growth inhibition}} - {\text{OD of minimum growth inhibition}}}}{{\text{OD of minimum growth inhibition}}}} \right] \times 100.$$

##### Morphological changes

At 40 and 100× magnifications, a light microscope was used to observe the morphological changes in cancer and normal cells treated with various concentrations of algal ChlE and ChlE-NS using acridine orange and ethidium bromide [AO/EB] [38].

##### DNA fragmentation assay

The DNA agarose gel electrophoresis method was used to evaluate the level of apoptosis that was induced in cancer cells. Cancer cells, namely, HEPG-2, MCF-7, HTC-116, and A549 were cultured in a plate with six wells for tissue culture and were treated with ChlE and ChlE-NS at the IC_50_% value at a concentration of 1 × 10^6^ cells/mL for each. Following a period of 24 h, the cells were collected with the use of a sterile scraper that contained 0.25% trypsin–EDTA. After collecting the cells, they were centrifuged for 10 min at a speed of 1000 rpm. After resuspending the collected pellets in a phosphate buffer solution, DNA was extracted using a DNA ladder detection kit (Biovision).

To lyse the cells, 5 μL TE lysis buffer was utilized. Following a 10-min incubation at 37 °C, 5 μL enzyme A solution was added to the cells, and the mixture was thoroughly combined using a gentle vortexing motion. The cells were then incubated at 50 °C for 30 min. After that, 5 μL ammonium acetate solution was added to each sample, and the mixture was completely combined. Next, 50 μL isopropanol was added and thoroughly mixed, and the cells were maintained at a temperature of – 20 °C for 10 min.

To precipitate DNA, each sample was centrifuged for 10 min and then dried in air for 10 min at room temperature. The supernatant was drained away, and the DNA pellet was washed in 0.5 mL of 70% ethanol. A 30 μL DNA suspension buffer was used to dissolve the DNA pellet. 15–30 μL of the sample was put on a 1.2% agarose gel that had 0.5 μg/mL of ethidium bromide in both the gel and the running buffer. A voltage of 5 V/cm was applied to the gel for one to two hours. The gel was visualized and photographed after 1.5 h under transmission UV illumination XD–79, WL/26 MX, 230 V50/60 HZ (Alliance 4.7, Taiwan, France).

##### Caspase-3 activity assay [39]

Cells were seeded in 6-well tissue culture plates (1 × 10^6^ cells/well) and incubated for 24 h before being treated with the IC_50_ value of *D. simplex* (ChlE and ChlE-NS) and incubated for an additional 48 h. Following that, the cells were collected and centrifuged at 1000 rpm for 10 min before being resuspended in (1×) PBS solution.

Except for the chromogen, 100 μL of the standard diluent buffer was added to the wells of the zero standard. The microtiter wells were then filled with 100 μL of controls or diluted samples. After mixing the diluted sample, the chromogen blank was left unfilled. The wells were covered and incubated for 2 h at room temperature, after which the solution was decanted from the wells and the liquid was discarded. The wells were then washed four times 100 μL of the active caspase-3 detection antibody solution was pipetted into each well (except the chromogen blank) and mixed. The wells were then incubated again for 1 h at room temperature, and the liquid was poured out and washed four times. In each well, 100 mL anti-rabbit IgG horseradish peroxidase (HRP) working solution was added (except in the chromogen blank). The plate was covered and incubated at room temperature for 30 min before being rinsed four times. Each well received 100 μL of stabilized chromogen.

During the time that the liquid in the wells was beginning to turn blue, a 100 μL stop solution was being carefully mixed into each well. As soon as 2 h had passed, the absorbance was measured at 450 nm against a chromogen blank made out of 100 μL of stabilized chromogen and stop solution.

#### Antioxidant activity

##### The scavenging assay of DPPH radical

The following procedure was used to perform the DPPH free radical assay [40]: In a 96-well plate (*n* = 6), 100 μL freshly made DPPH reagent (0.1% in methanol) was mixed with 100 μL of various concentrations of each sample (ChlE, ChlE-NS, or Trolox). The reaction was allowed to incubate for a full half an hour at room temperature and in the dark. At the end of the incubation time, the decrease in DPPH colour intensity was measured at 515 nm. Data are represented using the mean standard deviation (± SD). The percentage of inhibition was obtained using Trolox as the standard drug as follows (Eq. 3):

3 $$\mathrm{Inhibition} \left(\%\right)=\left[\frac{{A}_{1 }- A}{{A}_{1}}\right]\times 100,$$

where *A*_*1*_ is the reference absorbance and *A* is the sample absorbance. To determine the concentration of ChlE required to achieve a 50% decrease in the initial DPPH concentration, a curve of ChlE or ChlE-NS concentration vs DPPH scavenging activity (%) was created. This is referred to as the IC_50_.

##### ABTS-radical scavenging activity

The experiment was carried out in accordance with the approach described by Arnao et al. [41], but with a few minor adjustments to allow for its execution in microplates. Also, the reaction went better when 190 µL freshly made ABTS reagent was mixed with 10 μL of each sample (ChlE, ChlE-NS, or Trolox) at different concentrations in a 96-well plate. After a waiting period of ten minutes, the absorbance of the samples was measured using a microplate reader at 734 nm in comparison to the blank solution, which consisted of methanol. Every single experimental sample was performed three times over. The percentages of inhibition as well as the IC_50_ were determined (Eq. 4).

4 $$\mathrm{Inhibition} \left(\%\right)=\left[\frac{{A}_{\mathrm{control} }- {A}_{\mathrm{sample}}}{{A}_{\mathrm{control}}}\right]\times 100.$$

### Statistical analysis

The data are reported as the mean of three replicates' standard deviations (± SD). To determine whether the results were statistically significant, Tukey's multiple comparison tests using GraphPad Prism version 5 (GraphPad Software, San Diego, CA, USA) were used with one-way analysis of variance (ANOVA) to examine the collected data. A statistically significant difference was presumed to exist when *P* was less than 0.05.

## Results and discussion

### Preparation and characterization of ChlE

*D. simplex* is a type of red seaweed that is a member of the Rhodophyta phylum. It is one of the most widely distributed types of red seaweed along the coastlines of the Egyptian Red Sea, and it has demonstrated a variety of active metabolites that have the potential to act as possible therapeutic agents [28, 30]. It was reported that the kind of extraction solvent that was used had an impact not only on the chemical composition of these algal metabolites but also on the potential biological uses of these compounds [42]. Of these solvents, chloroform extracts of red marine macroalgae, in particular, are one of the natural resources for the production of essential bioactive secondary metabolites.

In this work, chloroform extract (ChlE) with a yield of 1.24% was made by extracting the dried powder of *D. simplex* after defatting it with petroleum ether. This extract was phytochemically screened, and the findings, shown in Table 1, indicated the presence of a variety of bioactive secondary metabolites, including terpenoids, phenolics, alkaloids, sterols, and polysaccharides. It is well known that phenolics and terpenoids possess powerful antioxidant and cytotoxic properties [22, 43–47]. Anthraquinones, flavonoids, saponins, and cardiac glycosides, on the other hand, were not present.

Because secondary metabolite isolation and identification is a time-consuming and resource-intensive procedure that requires both human and material resources, we adapted a rapid GC–MS study to perform chemical profiling as part of our research. A GC/MS analysis indicated the identification of 39 compounds, all of which belonged to various kinds of valuable and variable chemical classes. Table 2 summarizes the following information about these active metabolites: their names, retention times, molecular weights, molecular formulas, and area percentage. These bioactive compounds account for 87.48% of the total peak area. The oxygenated hydrocarbons were by far the most prevalent, accounting for 48.17% of the total compounds found, and these included esters (15.82%), ketones (4.96%), fatty acids (21.15%), and sterols (6.24%). The compounds that make up 37.02% of the total identified ones were hydrocarbons [straight (22.61%) and branched (13.32%) chains] and terpenoids (1.09%). There were also 2.29% of miscellaneous compounds. The primary fatty acid metabolite found in ChlE of *D. simplex* was 9-octadecenoic acid (oleic, omega-9 fatty acid) (16.47%). 2,3-Dihydroxypropyl (*Z*)-octadec-9-enoate (8.07%), 4-hydroxydecan-5-one (4.55%), cholesterol benzoate (3.57%), and fucosterol (2.67%) were found as the prevalent components in the other chemical classes. Previous research, including GC/MS analysis of seaweed extracts, supported similar findings [48–50].

**Fig. 1:**
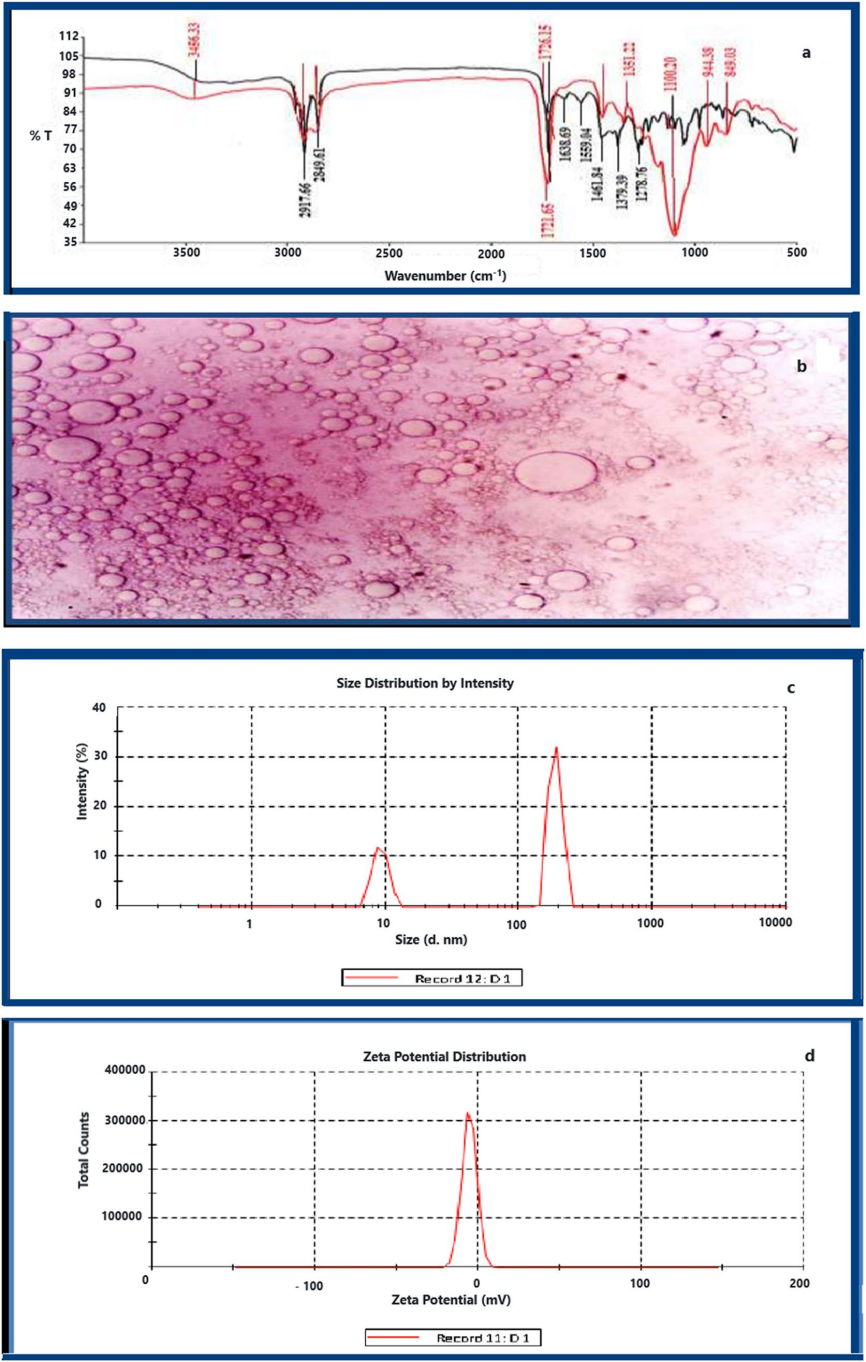
Characterization of ChlE-NS by: **a** FTIR analysis [ChlE, black curve & ChlE-NS, red curve], **b** light microscopy image, **c** particle size, and **d** zeta potential

An FTIR investigation of a *D. simplex* ChlE was carried out to get some insight into the possible existence of significant functional groups of bioactive chemicals in this extract. Figure 1 and Table 3 both include a listing of the individual bands, as well as estimates of their respective intensities and the functional categories that were found. These findings are in line with earlier research [51, 52].

**Table 3:** FT-IR absorption spectra and functional groups in *D. simplex* ChlE

Absorption frequency (cm^−1^)	Intensity estimation	Functional groups
3456.33	S	O–H, N–H stretching
2917.66	S	CH_2_ antisymmetric stretching of methyl groups
2849.61	S	C–H aldehydic
1721.65	S	C=O stretching
1638.69	W	C=C stretching
1559.04	W	C=C ( in ring) stretching
1461.84	M	S=O stretching/O–H bending
1379.39	M	CH_3_ bending
1278.76	W	NO_3_ stretching
1100.20	M	C–O stretching

*S* Strong, *M* Medium, *W* Weak

Figure 1a of the FTIR spectrum reveals that ChlE displays a strong peak at 3456.33 cm^−1^ owing to primary N–H group and primary O–H group vibrations. Strong peaks were observed at about 2917 and 2849 cm^−1^, and these peaks corresponded to the –CH_2_ antisymmetric stretch of methyl groups and C–H aldehydic groups, respectively. Also, a strong peak was found at 1721 cm^−1^ that matched the C=O stretching and a weak peak at 1638 cm^−1^ that matched the C=C stretching in aromatic molecules of phenyl and amide compounds, with a weak peak at 1559 cm^−1^ owing to aromatic C=C Stretching. Weak to moderate peaks were observed at 1461, 1379 and 1278 cm^−1^, and these peaks corresponded to S=O stretching/O–H bending, CH_3_ bend, and NO_3_ stretch, respectively. In addition, a moderate peak was found at 1100 cm^−1^ that matched the C–O–C group. It is worth noting that all of these peaks were reproduced in the ChlE-NS spectrum as well. This indicates that all key functional groups of ChlE were present in the final formulation of ChlE-NS, confirming the lack of extract–polymer incompatibility and ensuring that there were no chemical interactions between ChlE and any of the excipients utilized. These results are consistent with prior reports [53–55].

### Preparation and characterization of nanosuspension

The main problem with using chloroform extract to make a good dosage form is that it does not dissolve well in water. The best way to solve this problem and make these extracts more bioavailable is to use nanosuspension formulations. The goal of this study was to increase the bioavailability and solubility of ChlE by using a nano-precipitation technique to make the nanosuspension.

The antisolvent precipitation technique is one of the bottom-up approaches that may be used for the manufacture of nanosuspensions. This technique involves the precipitation of nanoparticles from a saturated extract solution. When compared to top-down approaches, the energy input required by this methodology is quite minimal [56]. To put it succinctly, ChlE is first dissolved in organic solvents [acetone and ethanol (3:1) by sonication], which is then quickly added into the antisolvent solution [water containing PVA 1.5% w/v] to precipitate the nanoparticles of ChlE due to its poor solubility in water with the assistance of a polymer (PVA) [57]. This method was seen as a simple, low-cost solution that could be used on a larger scale [58]. Electrostatic repulsion or steric effects may be achieved by adding tween 40 at the interface between the particles and the medium [59]. These effects prevent the particles from aggregating. Previous research [60, 61] concluded that a ratio of the drug to the stabilizer of 3:1 was the optimal option in terms of both performance and particle size. As a result, this ratio was employed, and it was determined to be the most effective choice. Light microscopy was used to examine the morphology of the produced formulation.

Figure 1b (light microscopy image) indicates that the produced particles were spherical in shape, with a distinct size distribution and no indication of aggregation. The particle size and polydispersity index (PDI) were determined using dynamic light scattering (DLS) on a Zetasizer Nano ZS from Malvern Instruments in the United Kingdom. The PDI was calculated to be 0.563, and the size distribution was determined to be 186.6 nm. The stability of the generated ChlE-NS given by PVA is shown in Fig. 1c. Hydrogen bonds were made between the functional groups of ChlE molecules and PVA molecules. This is what made this stabilization happen. As a consequence, Fig. 1d shows a reduced zeta potential (– 5.5 mV) [62], which might be attributable to PVA's nonionic stabilizing characteristics creating a coat around ChlE nanoparticles. As a surfactant, tween 40 is also important for making nanosuspension because it lowers the free surface energy of ChlE-NS molecules, which keeps them from sticking together [63].

### Cytotoxicity

**Fig. 2:**
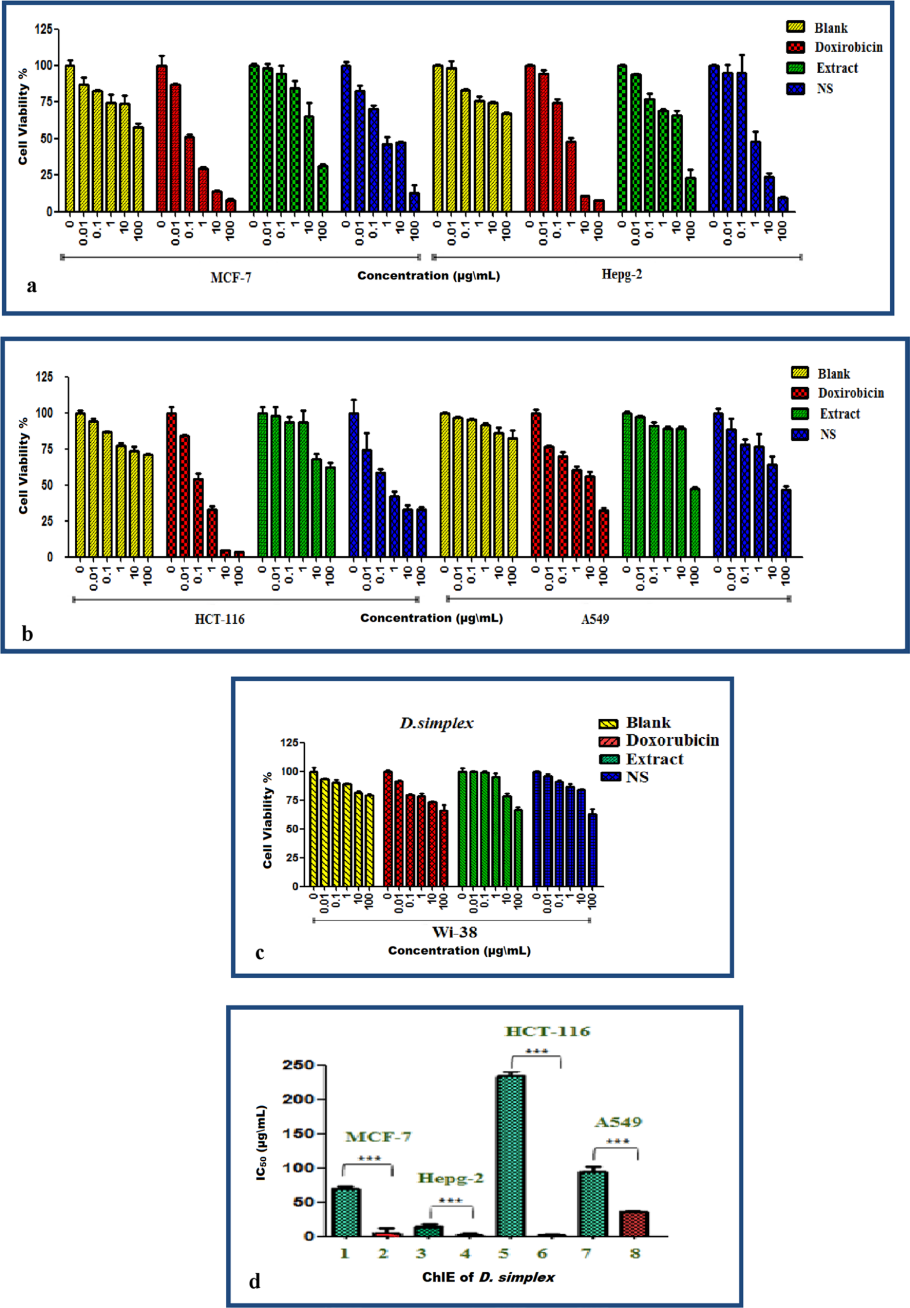
Bar graphs **a**, **b** & **c** represent dose-dependent cell viability percentage and bar graph d represents the significance of the IC_50_ of four cancer cell lines treated with *D. simplex* ChlE and ChlE-NS. The numbers 1, 3, 5 & 7 represent ChlE and 2, 4, 6 & 8 represent ChlE-NS. The data is expressed as the mean ± SD of three independent experiments. Statistical analysis consisted of an analysis of variance, *α* = 0.05 followed by a Tukey’s multiple comparison test. Tukey HSD: ns *P* > 0.05; *P ≤ 0.05; **P ≤ 0.01; ***P ≤ 0.001

*D. simplex* has been shown to have potentially useful phytoconstituents as cancer therapy agents [64]. Nanosuspension formulations improve the solubility of drugs that are only moderately soluble, which leads to a subsequent reduction in the drug dose while simultaneously successfully targeting specific cancer cells [65]. As a consequence of this, the purpose of this research was to investigate the cytotoxic potential of *D. simplex* ChlE and ChlE-NS formulations in relation to four different cancer cell lines. It was shown that, after 48 h of incubation with different concentrations of blank nanosuspension media (excipients without ChlE); IC_50_ values were more than 1000 µg/mL (Table 4 and Fig. 2a–d). The results of this experiment showed that PVA and tween 40, which were used as stabilizers and surfactants, had no effect on cell growth and could be used safely. An intriguing finding in the cytotoxicity trials was the impact of nanosuspensions (ChlE-NS). When compared to ChlE, ChlE-NS had superior effectiveness against all cell lines that were examined, in addition to having concentration-dependent cytotoxic effects. The cytotoxic activity was measured using the American Cancer Institute (NCI) protocol, which says that IC_50_ values of less than 20 µg/mL are important for crude extracts [66]. ChlE-NS had the most effective impact on HCT-116 cell lines, with an IC_50_ of 0.61 ± 13.8 µg/mL followed by HEPG-2 (1.37 ± 3.7 µg/mL) and MCF-7 (4.34 ± 6.4 µg/mL) cell lines in order of potency. In the meantime, a significant amount of activity was seen against A549 cell lines, with an IC_50_ value of 35.80 ± 5.9 µg/mL. Similar enhancements in cytotoxicity have been attributed to nanosuspension formulations for naringenin and other medicines [67, 68]. In contrast, ChlE had the most effective impact on HEPG-2 cell lines, with an IC_50_ of 13.08 ± 0.1 µg/mL, and moderate action on MCF-7 and A549 cell lines, with an IC_50_ of 69 ± 3.5 and 94.42 ± 1.5 µg/mL, respectively. It also had low activity on HCT-116 cell lines (IC_50_ = 234.9 ± 4.21 µg/mL). According to the aforementioned phytochemical investigation, ChlE was enriched with bioactive chemicals such as 9-octadecenoic acid (16.47%), fucosterol (2.67%), 2,3-dihydroxypropyl(Z)-octadec-9-enoate (8.07%) and others that have already been shown to be effective as cytotoxic agents [69–71]. Therefore, the cytotoxic effect of ChlE, which can be seen in the slower growth of cancer cells, is probably due to the presence of these bioactive compounds.

**Table 4:** Cytotoxic effects of *D. simplex* ChlE, ChlE-NS and blank (nanosuspension media without ChlE) against four human cancers and WI-38 normal cell line

Conc (µg/mL)	Cell viability (%)				
	MCF-7	HEPG-2	HCT-116	A549	WI-38
ChlE					
0	100 ± 2.41	100 ± 0.3	100 ± 4.4	100 ± 1.3	100 ± 3.1
0.01	97.9 ± 3.2	94.03 ± 0.4	97.6 ± 6.3	97 ± 0.8	99.6 ± 0.9
0.1	94.4 ± 2.5	76.9 ± 3.6	93.6 ± 3.4	90.7 ± 2.8	99.3 ± 1
1	84.7 ± 4.6	69.1 ± 1.2	93.4 ± 8.1	88.9 ± 1.2	95.6 ± 2.9
10	65.2 ± 0.23	66.2 ± 2.7	68 ± 13.7	88.9 ± 1.4	78.8 ± 1.9
100	31.5 ± 5.5	23.1 ± 6	62 ± 3.3	47.4 ± 1.2	66.3 ± 2.5
IC_50_ (µg/mL)	69 ± 3.5	13.08 ± 0.1	235.9 ± 4.21	94.42 ± 1.5	435.5 ± 2.7
ChlE-NS					
0	100 ± 1.3	100 ± 0.5	100 ± 9.4	100 ± 3	100 ± 0.6
0.01	82.9 ± 3.6	95.18 ± 5.4	73.9 ± 28.12	88.5 ± 7.2	95.9 ± 2
0. 1	70.1 ± 5.3	95 ± 12.6	58.4 ± 24.4	77.8 ± 3.8	91.2 ± 1.3
1	46.16 ± 4.5	47.8 ± 6.9	42.3 ± 20.8	76.5 ± 8.8	86.9 ± 2.08
10	47.6 ± 9.2	23.79 ± 2.4	33.1 ± 22.8	63.9 ± 5.7	84.3 ± 0.4
100	12.61 ± 0.8	9.8 ± 0.24	32.6 ± 26.2	46.5 ± 2.3	63.2 ± 3.9
IC_50_ (µg/mL)	4.34 ± 6.4	1.37 ± 3.7	0.61 ± 13.8	35.80 ± 5.9	852.4 ± 1.6
Doxorubicin					
0	100 ± 7.09	100 ± 0.55	100 ± 3.5	100 ± 1.69	100 ± 1.3
0.01	87.1 ± 0.6	94.16 ± 2.70	84.2 ± 0.6	76.47 ± 3.5	91.974 ± 0.3
0.1	51.03 ± 1.78	74.45 ± 2.90	54.29 ± 3.46	69.5 ± 2.33	79.5 ± 0.84
1	29.5 ± 1.105	48.27 ± 2.10	33.9 ± 1.9	60.23 ± 1.0	78.5 ± 2.73
10	14.9 ± 0.77	10.7 ± 0.28	4.69 ± 0.26	56.13 ± 0	73.4 ± 0.87
100	8.83 ± 1.21	7.7 ± 0.11	3.51 ± 0.2	32.22 ± 1.2	66.32 ± 4.57
IC_50_ (µg/mL)	0.18 ± 0.09	0.695 ± 0.06	0.18 ± 0.03	8.03 ± 0.06	703.5 ± 0.07
Blank (Nanosuspension media without ChlE)					
0	100 ± 3.62	100 ± 0.4	100 ± 1.6	100 ± 0.4	100 ± 0.98
0.01	86.9 ± 4.9	98.1 ± 4.9	94.8 ± 1.9	96.9 ± 0.2	98.6 ± 0.5
0.1	82.493 ± 1.05	83.1 ± 0.7	86.59 ± 0.8	95.4 ± 0.34	93.9 ± 3.8
1	74.4 ± 5.8	75.9 ± 3.1	77.5 ± 1.9	91.89 ± 0.7	90.7 ± 0.83
10	74.30 ± 5.4	74.9 ± 0.67	73.8 ± 2.56	86.3 ± 3.3	84 ± 0.74
100	77.93 ± 2.27	77.4 ± 0.7	70.94 ± 0.8	82.5 ± 5.3	83.7 ± 0.26
IC_50_ (µg/mL)	1341 ± 2.3	1424 ± 3.1	93,817 ± 1.9	17,373 ± 2.4	189,756 ± 0.7

**Fig. 3:**
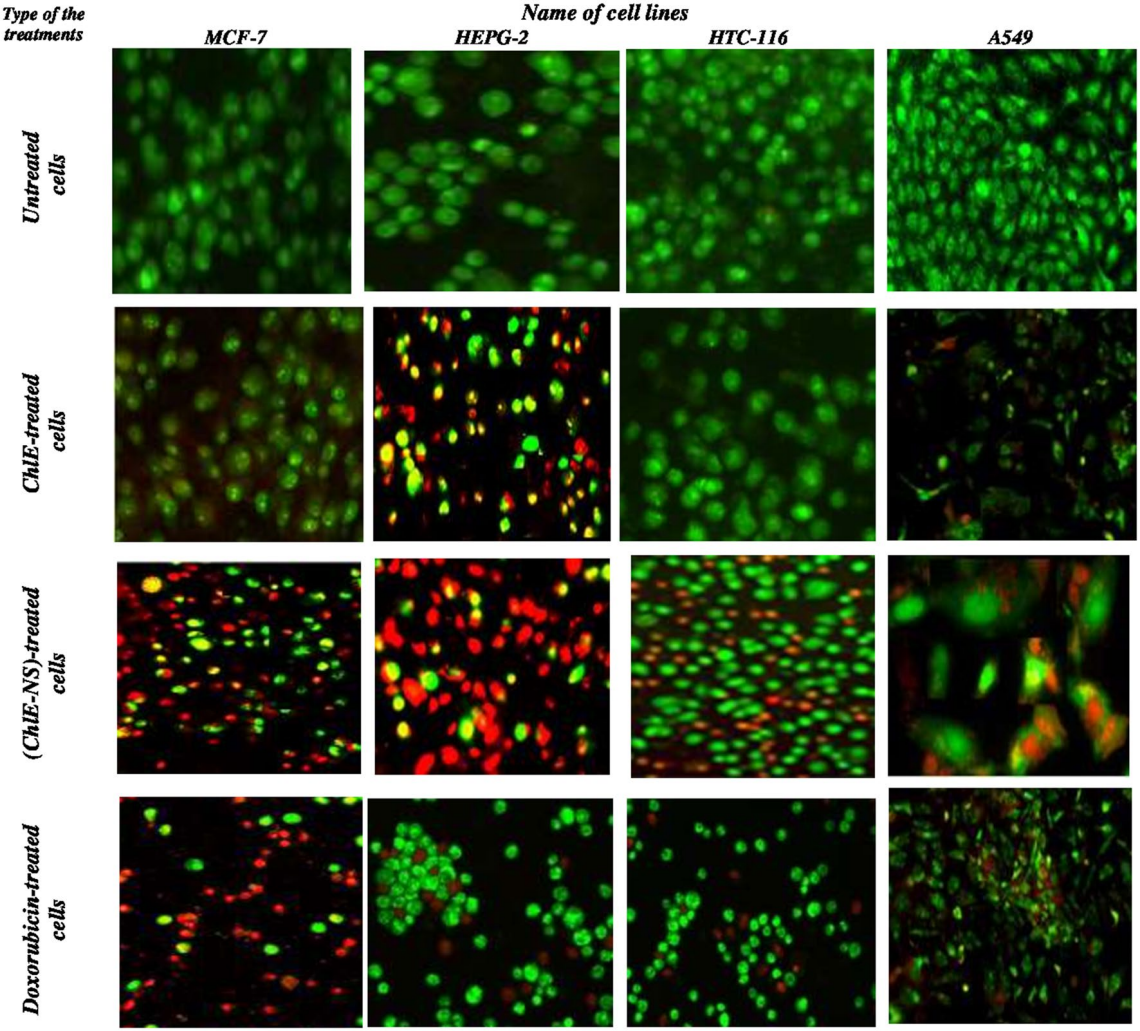
Morphological changes of untreated cancer and cancer-treated cells with ChlE and ChlE-NS at IC_50_ after AO/EB dual staining

**Fig. 4:**
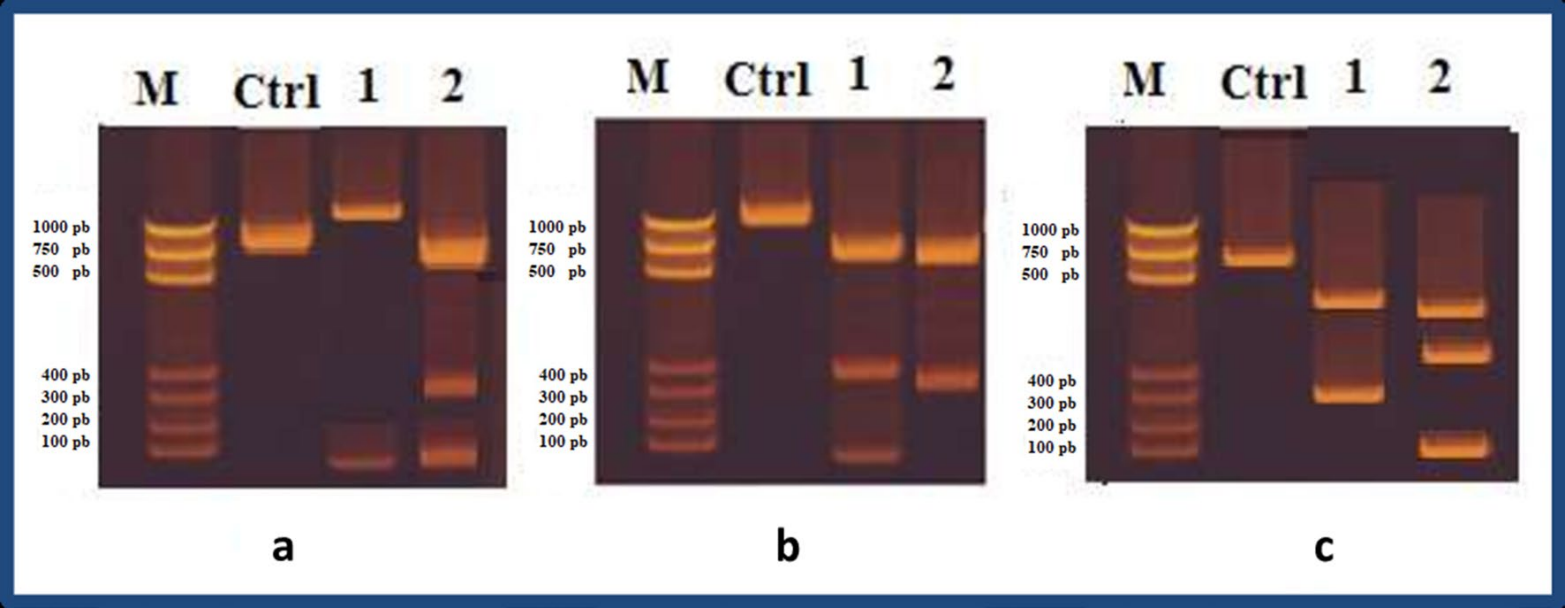
DNA fragmentation of: **a** = MCF-7, **b** = HEPG-2, and **c** = HCT-116cell lines. *M* Marker, *Ctrl* untreated cells, **1** = ChlE-treated cells, **2** = (ChlE-NS)-treated cells

For monitoring morphological changes of cancer cells, AO/EBr dual staining was chosen since it is simple to apply, inexpensive, and rapid. After labelling cancer cells treated separately with ChlE and ChlE-NS at IC_50_ for 48 h with AO/EBr, visible alterations were discovered. These changes included cell shrinkage, changed cell shape, and membrane blebbing, all of which are hallmarks of apoptotic cell death and are depicted in Fig. 3. AO stained all dead and living cells, whereas cells stained orange to red with EB when they lost their membrane integrity due to chromatin condensation and nuclear fragmentation [12], untreated cells (control) fluoresced green. According to the photos (Fig. 3), the cytotoxic effects of ChlE-NS were stronger than those of ChlE. These morphological alterations were detected in cancer cells when various nanosuspension formulations were used [72–75]. A DNA fragmentation assay was utilized to verify that cancer cells treated with *D. Simplex* ChlE and its nanosuspension suffered apoptosis as a direct consequence of DNA damage. Treatment with ChlE and ChlE-NS resulted in chromosomal DNA fragmentation in the MCF-7, HEPG-2, and HCT-116 cell lines. The second lane, which was treated with control cells, showed little fragmentation, whereas the third lane, which was treated with *D. Simplex* ChlE, showed considerable DNA fragmentation (Fig. 4). In contrast, Fig. 4 shows that the fourth lane, which was treated with nanosuspension, generates greater DNA fragmentation than the three other lanes.

**Fig. 5:**
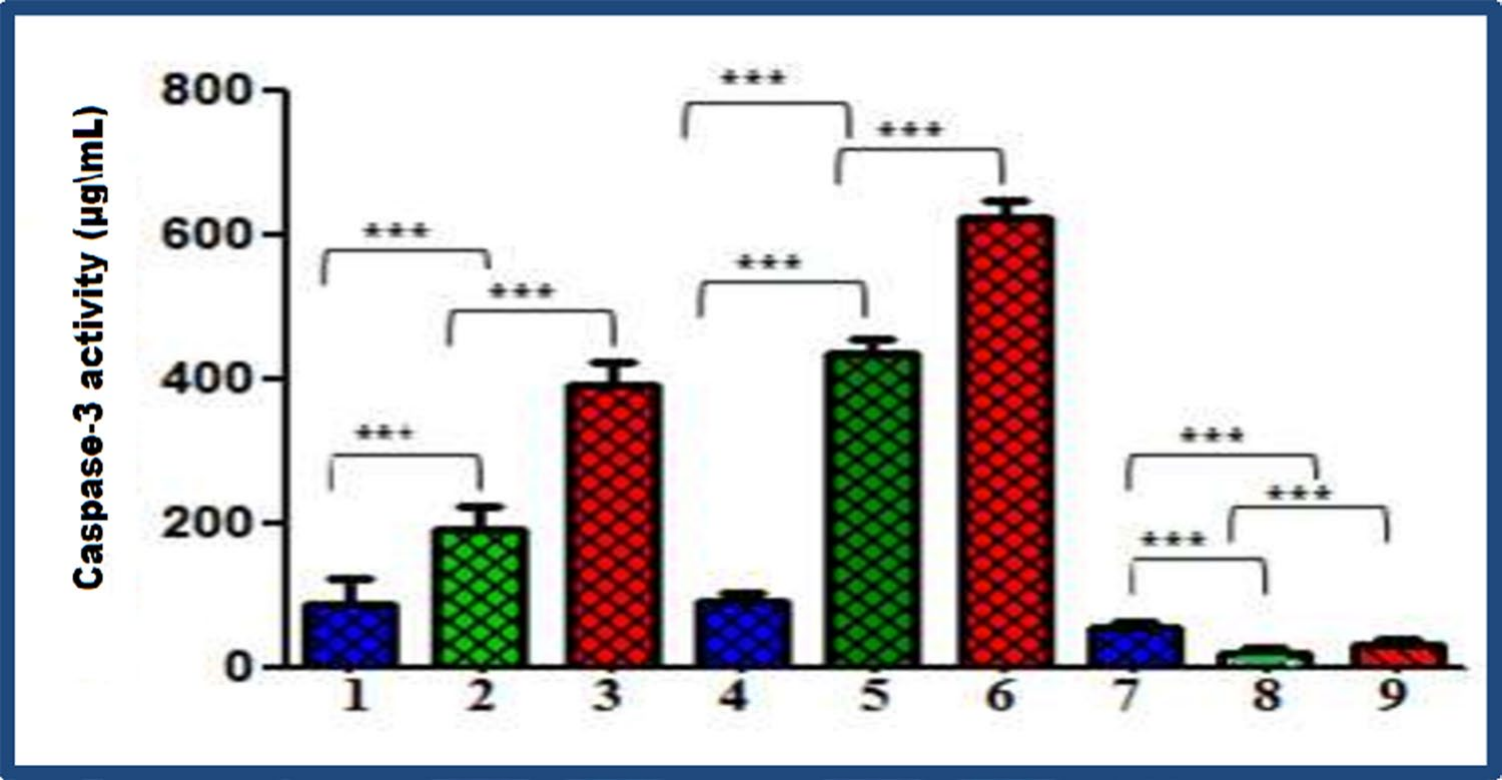
Bar graph illustrating the caspase-3 activity upgrade difference; 1, 4, 7 = MCF- 7, HEPG-2, HCT-116 cancer untreated cell lines; 2, 5, 8 = Cancer cells treated with ChlE; 3, 6, 9 = Cancer cells treated with ChlES of *D. simplex*. Statistical analysis of variance, α = 0.05 followed by a Tukey’s multiple comparison test. Tukey HSD: ns *P* > 0.05; *P ≤ 0.05; **P ≤ 0.01; ***P ≤ 0.001; ****P ≤ 0.0001

**Fig. 6:**
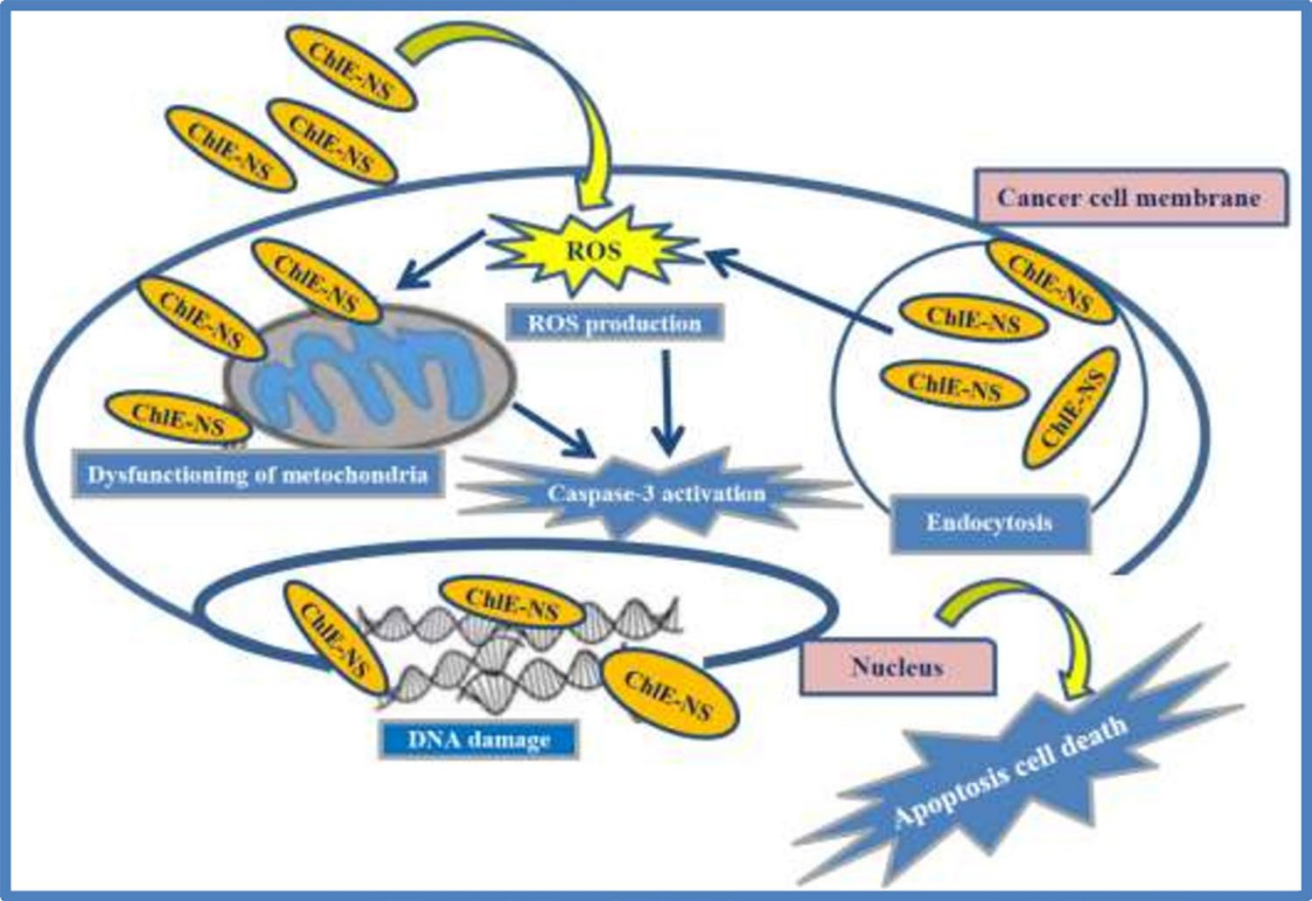
The proposed apoptotic mechanism of *D. simplex* ChlE and ChlE-NS

Both molecular pathways (intrinsic and extrinsic pathways) lead to the activation of caspase-3 and caspase-7. In this work, we followed the level of caspase-3 to gain insight into the molecular pathways of apoptosis, and the levels of caspase-3 were measured in MCF-7, HEPG2, and HCT-116 cells treated with *D. simplex* ChlE and ChlE-NS. Figure 5 demonstrates a substantial rise in the caspase-3 level in MCF-7 and HEPG-2 cells and a lower level in the case of HCT-116 cells relative to the control group (untreated cancer cells). In Addition, caspase-3 levels were significantly higher in ChlE-NS-treated cells than in ChlE-treated cells. In light of these findings, ChlE-NS proved to be more effective as an anticancer formulation than ChlE by inducing apoptosis, as evidenced by the activation of caspase-3 and the fragmentation of DNA. A schematic representation of the cell death mechanism is illustrated in Fig. 6.

The bioactive phytoconstituents of ChlE are maintained in the liquid phase via nanosuspensions, which are submicron colloidal molecules that are stabilized by the addition of stabilizers [76]. Also, there is a chance that the solubility and dissolution rates could be sped up, which would mean that there would be more of the drug near cancer cells [12, 71].

### Antioxidant activity

**Fig. 7:**
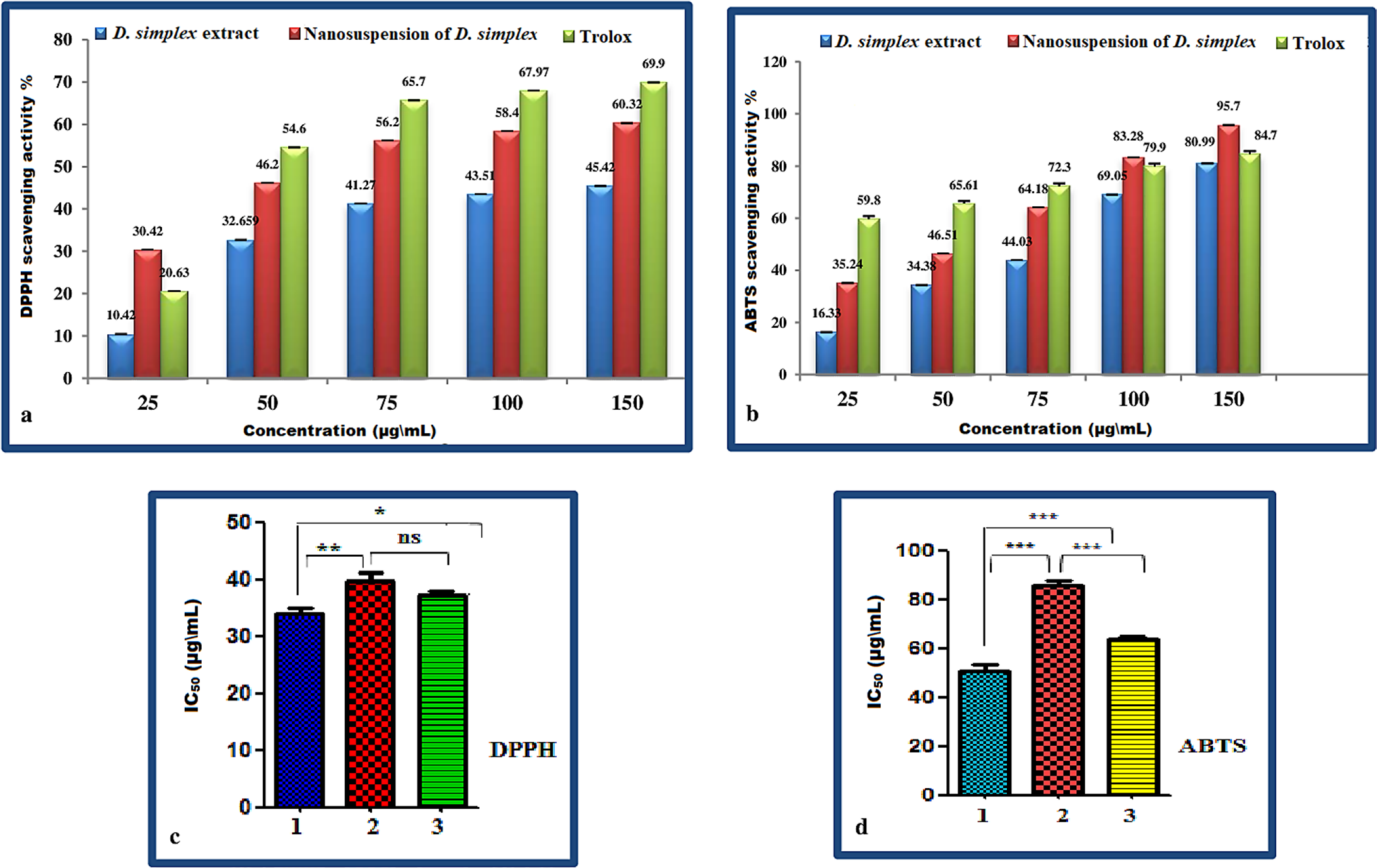
Bar graphs of DPPH and ABTS scavenging activity percentages (**a** & **b**) and IC_50_ significance (**c** & **d**) of the standard drug Torlox (1) and *D. simplex* ChlE (2) and ChlE-NS (3). Statistical analysis consisted in analysis of variance, *α* = 0.05 followed by a Tukey’s multiple comparison test. Tukey HSD: ns *P* > 0.05; *P ≤ 0.05; **P ≤ 0.01; ***P ≤ 0.001; ****P ≤ 0.0001

Natural antioxidants, such as those found in algae extracts, are gaining popularity as a potential alternative to synthetic antioxidants, which have been linked to a number of health problems. As a result, the DPPH and ABTS procedures were used to evaluate the antioxidant impact of ChlE and ChlE-NS [77]. These methodologies were chosen because of their low cost, high speed, and relative ease of use. The findings were shown in terms of percentage radical scavenging activity and IC_50_ (ug/mL) values in Table 5 and Fig. 7. Both ChlE and ChlE-NS had a concentration-dependent antioxidant impact, as shown by their antioxidant activity compared to ChlE, ChlE-NS exhibited a higher scavenging capacity for both DPPH and ABTS radicles. ChlE-NS outperforms ChlE because, by reducing particle size to the nano-range, the concentration gradient and surface area are enhanced, resulting in a significant increase in dissolving velocity [78]. A reduction in ChlE particle size has been shown to boost its in vitro antiradical potential, which might lead to an improvement in the in vivo activity.

**Table 5:** A comparison of the percentages of *D. simplex* ChlE and ChlE-NS scavenging activity at different concentrations using ABTS and DPPH to the reference drug, Trolox

Conc (µg\mL)	ABTS (% radical scavenging activity)			DPPH (% radical scavenging activity)		
	*D. simplex*		Trolox standard	*D. simplex*		Trolox standard
	ChlE	ChlE-NS		ChlE	ChlE-NS	
25	16.33 ± 0.4	35.24 ± 9.4	59.8 ± 4.8	10.42 ± 1.2	30.42 ± 9.3	20.63 ± 0.79
50	34.4 ± 1.15	46.51 ± 5.7	65.61 ± 5.1	32.7 ± 4.9	46.2 ± 5.3	54.6 ± 0.132
75	44 ± 3.66	64.2 ± 11.9	72.30	41.3 ± 8.3	56.2 ± 8.3	65.7 ± 0.32
100	69.05 ± 2.0	83.28 ± 5.8	79.9 ± 7.81	43.5 ± 8.0	58.4 ± 6.6	67.97 ± 0.68
150	80.99 ± 1.8	95.7 ± 7.5	84.7 ± 6.74	45.4 ± 7.9	60.32 ± 8.4	69.9 ± 0.90
IC_50_ (ug\mL)	86.5 ± 0.8	63.5 ± 0.47	49.38 ± 0.4	39.9 ± 0.08	36.86 ± 0.09	33.02 ± 0.6

## Conclusions

Chloroform extract-stabilized nanosuspension (ChlE-NS) of the red alga *Digenea simplex* was prepared for the first time by the antisolvent precipitation method using PVA (as a stabilizer). The chemical profile of the chloroform extract (ChlE, the crude extract) was evaluated by phytochemical screening tests, Fourier transform infrared spectroscopy (FTIR), and gas chromatography/mass spectroscopic analysis (GC/MS). The in vitro antioxidant and anticancer properties of the ChlE and the prepared NS were compared. An apoptotic mechanism was established using acridine orange/ethidium bromide (AO/EB) dual staining, DNA fragmentation, and increased caspase activity. The anticancer and antioxidant activities of the nanosuspension formulation were found to be significantly higher than those of the crude extract. This may be because nanosuspension keeps bioactive phytoconstituents of ChlE in a liquid phase as submicron colloidal molecules are stabilized by the added stabilizers. As a result, the surface area increased, resulting in enhancement in bioactivities. In the future, in vivo studies will be needed to confirm that the current nanosuspension formulation has the most therapeutic evidence and to evaluate its clinical efficacy.
